# More coercion, less support: A latent class analysis of post-incident reviews for mental health inpatients exposed to coercive practice

**DOI:** 10.1371/journal.pmen.0000511

**Published:** 2026-04-15

**Authors:** Lewys Beames, Kia-Chong Chua, Alan Simpson, Juliana Onwumere

**Affiliations:** 1 Department of Psychology, Institute of Psychiatry, Psychology & Neuroscience, King’s College London, London, United Kingdom; 2 Bethlem Royal Hospital, South London and Maudsley NHS Foundation Trust, Beckenham, United Kingdom; 3 Biostatistics and Health Informatics, Institute of Psychiatry, Psychology and Neuroscience, King’s College London, London, United Kingdom; 4 Centre for Mental Health Nursing Research, Care in Long Term Conditions Research Division, Florence Nightingale Faculty of Nursing, Midwifery and Palliative Care, King’s College London, London, United Kingdom; 5 Health Service and Population Research, Institute of Psychiatry, Psychology and Neuroscience, King’s College London, London, United Kingdom; PLOS: Public Library of Science, UNITED KINGDOM OF GREAT BRITAIN AND NORTHERN IRELAND

## Abstract

In psychiatric inpatient care, coercive practices (e.g., physical restraint) are strategies employed to support assessment, treatment and safety plans. Their use, however, is associated with physical and psychological harms. Clinical guidelines recommend offering patients a post-incident review to mitigate these harms, yet evidence suggests these are not routine or consistently implemented. Understanding variation in their provision across coercive events and patient groups, is essential for developing effective and equitable post-coercive practice support interventions. This study aimed to identify distinct profiles of coercive practice exposure among mental health inpatients and examine how these profiles, alongside demographic factors, relate to use or omission of post-incident reviews. A cross-sectional, retrospective analysis of three years of anonymised patient incident data (>8,000 incidents, ~1,600 patients) from the centralised electronic incident reporting system of a mental health service provider in England was conducted. Latent class analysis and multinomial regression examined associations between class membership post-incident review occurrence and staff-reported reasons for omission. Latent class analysis identified four profiles: (1) removal/ separation, (2) threat-compliance coercion, (3) passive refusal, and (4) resistive refusal. Profiles were interpreted as differing in relative invasiveness and restrictiveness based on the types and combinations of coercive practices. Post-incident reviews were significantly less likely to occur following more invasive exposures. Black and racially minoritised groups were more frequently represented in profiles less likely to receive a post-incident review. Profile membership also predicted staff-reported reason for omission of post-incident review. Variation in implementation of post-incident reviews in inpatient mental healthcare is influenced by the coercive practice context and demographic factors. Findings suggest that broadening the scope of post-coercive practice support and tailoring it to the specific coercive practice context may enhance patient experience and help address inequities.

## Introduction

### Coercive practice in mental healthcare

Each year, approximately 3.25 million people in England access mental health services with 3% (~97,500) requiring a psychiatric inpatient admission [1,2]. Over half of these admissions are involuntary, meaning patients can be assessed and treated without their consent [3]. In the United Kingdom (UK) coercive practices, including involuntary admission, physical restraint and forced medication, are legally employed within mental health care provision and legislation (e.g., Mental Health Act (1983, 2005)) to support assessments and treatments, and maintain patient safety [4]. Coercive practices raise complex ethical and clinical issues for psychiatric staff [5–7]. They are associated with physical and psychological harms for patients, families/carers, and staff [5,8] including physical injuries and Post-traumatic Stress Disorder [6,7,9]. They can also damage patient-staff relationships, [10] patient engagement with services, recovery trajectories, and overall patient outcomes [11]. Furthermore, people from Black and racially minoritised ethnic groups, are exposed to disproportionately greater levels of coercive practice [2] with evidence confirming racial bias in risk assessments [12].

### Post-incident reviews

To mitigate harms, clinical guidelines [13] and legislation, (e.g., Mental Health Units (Use of Force) Act (2018) in England), recommend offering post-incident reviews to patients exposed to coercive practice. Post-incident reviews, also termed ‘debriefs’, post-seclusion and/or restraint review, and post-occurrence reviews, [14,15] are a structured intervention intended to provide emotional support to the patient, generate staff learning from the incident, and contribute to the reduction of further coercive practice [16]. The reviews are included in established multicomponent restraint reduction interventions including Six Core Strategies [17] and Safewards, [18] and have been evaluated as a standalone intervention [19,20]. However, post-incident reviews lack definitional clarity and vary in thier operationalisation including their purpose, components, timing, and participants [15,20]. Hospital policies provide limited guidance for staff in how to conduct post-incident reviews, [16] for example, the relevant policy for the mental health service provider assessed in the present study does not delineate patient and staff procedures, prescribe timeframes, or indicate procedures around review omission [21].

Notwithstanding policy recommendations, post-incident reviews are not consistently offered, with prevalence estimates ranging from 27-93% [19,22]. Qualitative evidence from staff and patients also suggests they are neither routine on mental health wards nor consistently implemented and in some cases, ineffective delivery is experienced as a continuation of the coercive experience [23–25]. Further research is needed to understand variation in post-incident review provision including the factors associated with their occurrence and non-occurrence. This would inform intervention development and implementation strategies to improve consistency in applications and, potentially, its effectiveness.

Across clinical and research contexts coercive practices are often considered in isolation. In contrast, patient experiences are defined by the cumulative and interrelated nature of these individual forms of coercion [8]. Latent class analysis is a statistical method to identify unobserved subgroups in a population based on categorical data [26]. In this study latent class analysis was used to model the latent construct of ‘coercive practice exposure’. This allowed for the identification of clinically meaningful profiles based on patient’s cumulative experiences of multiple coercive practice occurring within a singular incident. This approach also accommodates the inclusion of covariates, enabling examination of how incident and demographic factors relate to the occurrence and non-occurrence of patient post-incident reviews.

This study aims to:

i Identify distinct coercive practice exposure (incident) profiles in mental health inpatients through a Latent Class Analysis of inpatient incident data from a large NHS mental health service provider.
ii Examine how the identified profiles, alongside demographic factors, relate to implementation or omission of post-incident reviews in inpatient mental healthcare.

## Method

### Ethics statement

Approval to access the fully anonymised dataset was received by the South London and Maudsley NHS Foundation Trust’s committee for clinical evaluations and projects (PPF2023248). This study complies with the ethical standards of the relevant national and institutional committees and with the Helsinki Declaration of 1975, as revised in 2013.

### Design

A cross-sectional design using retrospective analysis of three years of anonymised incident data from a large mental health service provider.

### Patient and public involvement and engagement

A Lived Experience Advisory Panel (LEAP) was established to support the study (Reported according to the GRIPP-2 short-form in Table A in S1 Text). The panel comprised people with direct and indirect experience of coercive practice in inpatient mental healthcare as a patient or family/carer. The panel contributed to the interpretation and naming of latent class profiles, and to the interpretation of the regression analysis.

### Setting

The mental health service provider is one of the largest mental healthcare providers in Europe and delivers services across the life course, including acute admission, forensic wards, and a range of national and specialist services (e.g., an eating disorders unit). The service provider serves an ethnically and socially diverse population of approximately 1.4 million people across four London boroughs [27]. Approximately 5,000 people receive inpatient care annually across the service provider’s, 4 hospitals and 47 wards.

### Data sources

Coercive practice incident reports manually completed and submitted by staff on a centralised electronic reporting system, titled ‘Datix’. Datix is a web-based incident reporting system used by several NHS hospitals to report incidents. The system comprises data on all reported uses of coercive practice and limited demographics (i.e., age, gender, ethnicity). Diagnosis and other clinical data are not available in the dataset. Since March 2018 data on post-incident reviews has been included. A unique incident ID number is assigned to each incident while individuals are identified by a case ID number. The fully anonymised dataset was accessed on 18^th^ August 2023.

### Sample

Data comprised incidents occurring over a 3-year period [1^st^ January 2020 to 31^st^ December 2022]. This timeframe was selected due to changes in the reporting system, including the introduction of a new post-incident review field and a major system upgrade, that made earlier data incompatible.

Inclusion criteria: Incidents with a unique incident ID in an inpatient setting, involving at least one patient with a unique case ID where ‘role in the incident’ (a field in the dataset defining how an individual was involved in an incident) was recorded as ‘patient who was restrained’, ‘perpetrator’, ‘involved’ or ‘person affected/ injured’ and where coercive practice was recorded as ‘yes’.

Exclusion criteria: Incidents outside of the study period with no unique incident ID or case ID occurring in non-inpatient settings (community team, a non-mental health hospital, prison, private hospital) or involving only ‘victim’, ‘witness’ or ‘staff’ as the ‘role in the incident’.

For incidents with multiple cases, the case assumed to be subject to coercive practice was selected using the following ‘role in incident’ hierarchy: ‘patient who was restrained’, ‘perpetrator’, ‘involved’ or ‘person affected/ injured’. Patients where all cases had the same ‘role in incident’ were excluded.

To account for repeated cases within the data the unit of analysis was the incident level, rather than the patient level, such that each incident was treated as a single observation in the analyses.

### Variables

Primary outcomes were occurrence of patient post-incident review and reason for non-occurrence. The variable ‘*Did a patient post-incident review occur*?’ was recorded as ‘Yes’, ‘No’ or ‘Not recorded’ (recoded missing data). If ‘No’ was selected, staff select a reason from a drop-down menu: ‘Patient refused to take part’, ‘Clinical decision to delay’, or ‘Other’. These are the only variables in the dataset that concern post-incident reviews, and completion of these fields is mandatory in Datix. The relevant mental health service provider’s policy does not define these terms [21].

Exposure variables (Table B in S1 Text) consist of the coercive practices reported in each incident. Data are reported as broad categories and specific types within these categories. For example, ‘physical restraint’ includes different restraint positions and physical holds (e.g., ‘standing’, ‘prone’). To facilitate analysis, coercive practice variables were coded numerically, ‘1’ (Yes) or ‘2’ (No) indicating whether it was used in the incident.

Covariates comprised patient main demographics: age, gender, and ethnicity. Age was recorded as discrete data with no transformations. Gender was recorded in the raw data as: ‘Female’, ‘Male’, ‘Transgender’ and ‘Not specified’. Due to low counts (<5) in the ‘Transgender’ category, data were merged with ‘Not specified’ to preserve anonymity. Missing data were recoded as ‘Not recorded’.

Ethnicity was reported across eighteen categories with some yielding low frequencies (e.g., Chinese (n = 8), Pakistani (n = 4)). To facilitate analysis and aid interpretability, ethnicity data were recoded to UK Census 2021 [27] high-level groups, ‘Asian’, ‘Black’, ‘Mixed’, ‘Other’, ‘White’, plus ‘Not stated’ (i.e., patient choice not to provide this data) and ‘Not recorded’ (recoded missing data).

### Statistical analysis

Analyses were conducted using RStudio version 4.3.1 [28] employing the poLCA_1.6.0.1 package for the Latent Class Analysis.

Descriptive statistics summarised incident and case characteristics, and cross-tabulations were used to explore relationships between categorical variables.

#### Latent class analysis.

The three-step latent class analysis approach was used [26,29,30] as it benefits from reduced risk of misclassification and greater clarity of the effect of covariates on the latent variable of interest [29].

One-class models were first estimated, with additional classes added to assess if they improved the model. Improvement in model fit was evaluated using multiple fit criteria [26,30]; smaller Akaike Information Criterion (AIC), Bayesian Information Criterion (BIC), and sample-adjusted BIC (SABIC) values indicate better fit. Bootstrapping was conducted to assess whether improvements were statistically significant, as indicated by p-values smaller than 0.05 for Lo-Mendell-Rubin likelihood ratio test (LMR LRT) and the bootstrap likelihood ratio test (BLRT). In addition, good classification is indicated by entropy values of 0.8 or higher. Qualitative interpretability of the models was reviewed by the research team, the LEAP, and an external coercive practice research group. As the dataset did not include a measure of degree of invasiveness or restriction, interpretations of the classes were based on the types and combinations of different types of coercive practice, drawing on existing evidence, [31] lived experience perspectives, and clinical understanding of the relative invasiveness and restrictiveness of different types of coercive practice in isolation and combination.

Models that demonstrated the best fit indices (e.g., AIC, BIC) and diagnostic criteria (e.g., LMR LRT, entropy) and were clinically interpretable were taken forwards. Each incident was assigned to a class based on the posterior probabilities of the latent class analysis model. Measurement parameters of the latent class analysis model were then fixed. To account for individual cases being associated with multiple incidents in the dataset, each incident was treated as a unique observation and posterior probabilities applied at the incident level. Covariates were added to the latent class analysis and the models evaluated using the same fit indices, diagnostic criteria and qualitative interpretability considerations as in step 1.

Cases with missing data were included at step 1, estimation of the model, but were automatically removed by the poLCA package in step 3 (addition of covariates), reducing the total number of incidents included.

#### Regression analysis.

Two multinomial regression models examined whether latent class, as derived from relevant incident and individual factors, predicted, i) post-incident review occurrence and ii) reason for non-occurrence. In both models, latent class was included as the predictor variable. Outcome variables were ‘Yes’, ‘No’ and ‘Not recorded’ for the first analysis, and ‘Yes’, ‘Patient refused to take part’, ‘Clinical decision to delay’, ‘Other’ and ‘Not recorded’ for the second. ‘Yes’ was selected as the reference category as it was the largest category in both analyses. Descriptive statistics described the age, gender and ethnicity of each class.

## Results

The dataset contained 8,263 incidents, from 47 inpatient wards, over the three-year period with 1,599 cases (i.e., patients exposed to coercive practice). 99% of incidents contained complete data on occurrence of patient post-incident reviews. Of these a review was reported in 84% (n = 7206) of events while in 15% (n = 1288) of events, no post-incident review occurred.

Table 1 presents the frequency of patient post-incident review by age, gender, and ethnicity. Asian (89%) or Mixed (87%) patient ethnicity were associated with highest frequency of post-incident reviews. Occurrence was close to equivalence for women (83%) and men (80%). Table 2 presents the frequency of reported reasons for non-occurrence of patient post-incident review by patient demographics.

**Table 1:** Frequency of patient post-incident review by demographic factors (gender, age and high-level ethnicity).

Characteristic	Yes	No	Not Recorded	Total (%)
**Gender, n (%)**				
Male	713 (79.84)	172 (19.26)	8 (0.9)	893 (55.85)
Female	577 (82.9)	115 (16.52)	* (0.57)	696 (43.51)
Not specified	10 (100)	0 (0)	0 (0)	10 (0.63)
**Age (years)**				
Mean (SD)	35.3 (15.2)	38.8 (18.0)	40.6 (14.6)	36.24 (15.55)
Median	32	35	42	33
IQR (Q1-Q3)	21 (24-45)	26 (25-51)	27.2 (26.5-53.8)	22 (24-46)
Recorded				1476 (92.31)
Not recorded				123 (7.69)
Ethnicity**, n (%)				
Not recorded	351 (79.59)	89 (20.18)	* (0.23)	441 (27.58)
Asian	39 (88.64)	5 (11.36)	0 (0.00)	44 (2.75)
Black	185 (78.72)	47 (20.00)	* (1.28)	235 (14.7)
Mixed	41 (87.23)	* (10.64)	* (2.13)	47 (2.94)
Not stated	232 (80.00)	54 (18.62)	* (1.38)	290 (18.14)
Other	324 (83.72)	60 (15.50)	* (0.78)	387 (24.2)
White	128 (82.58)	27 (17.42)	0 (0.00)	155 (9.69)

*<5 - value not reported to preserve anonymity.

**Higher order ethnicity categories as per UK Census definitions (36).

SD: Standard Deviation.

IQR: Interquartile range; Q1 = lower quartile, Q3 = upper quartile.

**Table 2:** Frequency of reported staff reason for post-incident review non-occurrence by demographic and incident factors.

Characteristic	Clinical Decision to Delay	Other	Patient Refused to Take Part	Total
**Gender, n**				
Male	59	46	146	251 (56.15)
Female	29	40	127	196 (43.85)
**Age (years)**				
Mean (SD)	32.36 (16.1)	37.8 (16.7)	41.5 (18.5)	39.28 (18.34)
Median	30	36	37	36
IQR (Q1-Q3)	20.8 (20.2-41)	21 (25-46)	29.5 (25.5-55)	27.00 (25.00-52.00)
Min-Max	14-84	18-82	15-87	14-89
Ethnicity**, n				
Not recorded	27	22	41	90 (31.25)
Asian	*	*	0	5 (1.74)
Black	13	7	27	47 (16.32)
Mixed	*	0	*	* (1.74)
Not stated	10	8	36	54 (18.75)
Other	10	11	39	60 (20.83)
White	*	6	19	27 (9.38)

* <5 – value not reported to preserve anonymity.

**Higher order ethnicity categories as per UK Census definitions (36).

SD: Standard Deviation.

IQR: Interquartile range; Q1 = lower quartile, Q3 = upper quartile.

### Latent class analysis

Model fit criteria indicated a seven-class model (Tables C and D in S1 Text). However, AIC and BIC values plateaued from the 3-class model suggesting only marginal improvements in model fit with increasing number of classes. Models 3–7 were therefore deemed acceptable on these criteria. Class sizes reduced to 7–8% for models 4–7 although remained within acceptable limits [26]. LMR LRT and the BLRT were significant with the addition of each class indicating model fit was significantly improved by the additional class. Although not used for final model selection, it should be noted, however, that only 3 and 4 class models had adequate entropy (above 0.8) suggesting inferior clarity of the classifications in models 5 and onwards. Following examination of the interpretability of the models by the research team, LEAP and external research team, models 3 through 6 were taken forwards. Following the addition of covariates to the latent class analysis and re-evaluation against fit criteria, diagnostic criteria (Table 3) and assessment of interpretability the 4-class model was selected as the final solution.

**Table 3:** Latent Class Model Fit and Diagnostic Criteria.

nclass	nsample	Observed cases	Npara-meters	LL	AIC	BIC	SABIC	Smallest class count (n)	Smallest class size (%)	LMR LRT (p value)	BLRT (p value)	Entropy
3	7901	7812	79	-42198.89	85035.63	85586.63	85339.13	2593	0.33	–	–	0.87
4	7901	7812	109	-41625.33	83943.04	84703.28	84361.79	1084	0.13	0.00	<0.001	0.92
5	7901	7812	139	-44664.34	88764.27	89733.76	89298.27	1	0.00	1.00	1.00	0.99
6	7901	7812	169	-44588.96	88207.73	89386.46	88856.98	0	0.00	0.00	<0.001	-NaN

#### Final model.

**Fig 1:**
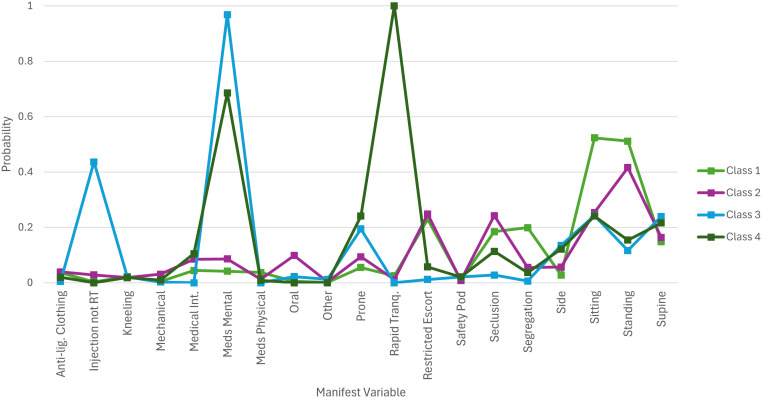
4-Class Model Including Covariates, Age, Gender and Ethnicity.

Fig 1 is a plot of the final 4-class model including covariates. Age, gender and ethnicity composition of the classes is reported in Table E in S1 Text.

Class 1 – ‘Removal/ separation’ – comprised 34% of the sample and was characterised by physical restraint in sitting and standing positions, restrictive escort (physical restraint employed to move the patient), seclusion and segregation and a very low probability of medications or chemical restraint being employed. The profile appears to describe a scenario in which an individual is temporarily removed from a situation, i.e., a behavioural time-out. This class was predominantly female (81%), of young age (18 years or under) and of White, Not stated or Not recorded ethnicity.

Class 2 – ‘Threat-compliance coercion’ - contained 13% of the sample and, like Class 1, included moderate to high probabilities of a range of physical restraint positions. What distinguishes this class, however, is a higher probabilities of oral chemical restraint, forced medical intervention and low probability of segregation. This class appears to describe the scenario of a team of staff approaching a patient to force them to take medication or other treatment and as such was labelled ‘Threat-compliance coercion’. This class included a larger proportion of men, wide variation in age with a mean of 33 years (sample mean 36 years) and a higher proportion of patients of Other, Not stated and Black ethnicity. Low proportion of patients of Asian and Mixed ethnicity.

Class 3 – ‘Passive refusal’ - included 27% of the sample and is defined by high probability of forced psychiatric medications (treatment medications, e.g., anti-psychotic depot) in the absence of chemical restraint (sedating medications) or other measures that may be used to mitigate risk of violence. The absence of such risk mitigation measures suggests patients in this class are not accepting of treatment willingly but are not physically resisting it. This class has the oldest mean age, gender is approximately evenly distributed, high proportion of patients of Black and Other ethnicity. Moderate proportion patients with missing ethnicity (Not stated, Not recorded) ethnicity. White, Mixed and Asian ethnicity is notably low in this class.

Class 4 – ‘Resistive refusal’ – comprising 25% of the sample, was demarcated by a high probability of forced psychiatric medications in combination with rapid tranquillisation and a low to moderate probability of seclusion. This class appears to describe the clinical scenario in which a patient is forcibly administered medication by injection and where staff anticipate a risk of, or encounter actual, physical resistance from the patient, leading to the use of more restrictive and intrusive coercive practices. The mean age for this class was 36 years, and it comprised a higher proportion of women. Patients with Other ethnicity were overrepresented in this class, with moderate proportions of those recorded as Not stated, Not recorded and Black ethnicity. In contrast, the proportion of White, Mixed and Asian ethnicity patients in this class was low.

### Predictors of post-incident review

Latent class significantly predicted whether post-incident review was reported as ‘No’ compared to ‘Yes’ (β = 0.3084, SE = 0.0271, p < 0.001). Odds ratios indicate that for every unit increase in latent class (i.e., progression from class 1 through to 4) odds of post-incident review being recorded as ‘No’ increased by 36.1% (OR = 1.3612, 95% CI [1.291, 1.435]).

Latent class also significantly predicted reporting of post-incident review as ‘Not recorded’ compared to ‘Yes’ (β = 0.2609, SE = 0.0912, p = 0.0042). Odds ratios indicate that for each increase in class, odds of ‘Not recorded’ being reported increase by 29.8% (OR = 1.2982, 95% CI [1.086, 1.552]).

Latent class was a significant predictor of ‘patient refused to take part’ as reason for no reported post-incident review (β = 0.4173, SE = 0.034, p < 0.001). For each increase in class, odds of ‘patient refusing to take part’ being reported as the reason for no post-incident review increased by 51.8% (OR = 1.518, 95% CI [1.420, 1.622].

Latent class was not a significant predictor of ‘clinical decision to delay’ as the reported reason for no post-incident review (β = 0.0394, SE = 0.0556, p = 0.4792, OR = 1.04, 95% CI [0.933, 1.160]).

The reason ‘Other’ was significantly predicted by latent class (β = 0.2496, SE = 0.0616p < 0.00005) with the odds of ‘Other’ being reported as the reason for omission of post-incident review increasing in each successive class by 28.4% (OR = 1.284, 95% CI [1.138, 1.448]).

Finally, latent class significantly predicted ‘Not recorded’ as reason for no post-incident review (β = 0.26, SE = 0.0913, p = 0.0044). For each increase in latent class, odds of ‘Not recorded’ being the reported reason for review omission increased by 29.7% (OR = 1.297, 95% CI [1.085, 1.551]).

## Discussion

This study aimed to identify distinct profiles of coercive practice exposure among mental health inpatients and examine how these profiles, alongside demographic factors, relate to use or omission of post-incident reviews. Analysis of an electronic incident dataset (>8000 incident, ~1600 patients) yielded four distinct coercive practice exposure profiles: 1. removal/separation, 2. threat-compliance coercion, 3. passive refusal, and 4. resistive refusal.

The results offer three main observations. First, while post-incident reviews were reported to have occurred in most incidents, their frequency varied across coercive practice exposure profiles. Post-incident reviews were less likely in profiles characterised by more invasive or restrictive types of coercive practice such as, physical restraint and forced medication. Second, profiles characterised by more invasive and restrictive coercive practices contained a greater proportion of patients from Black and racially minoritised groups. Third, reasons reported by staff for review omission varied across the four profiles with ‘refused to take part’, ‘other’ and ‘not recorded’ more common in profiles with more invasive or restrictive types of coercive practice.

### Coercive practice exposure profiles

The four exposure profiles identified in this study varied in coercive practice type, and demographic composition. Based on the types and combinations of coercive practices, profiles were interpreted post-hoc as differing in relative invasiveness and restrictiveness. Class 1 primarily included less invasive forms of coercive practice, while other classes contained higher probabilities of coercive practice generally considered to be more invasive and restrictive (e.g., forced medication by injection). Class 4 contained the highest proportion of the more invasive and restrictive practices.

The ‘removal/ separation’ class exclusively comprised young people (<18 years) and resembles a behavioural ‘time out’ strategy in which individuals are temporarily removed from situations thought to be promoting or reinforcing undesirable behaviours [34]. This may reflect a conceptually distinct approach to coercive practice in child and adolescent services. Although less invasive than other profiles, from a trauma informed perspective, ‘time-out’ may be seen as punitive [35] and risk re-traumatisation [36]. However, proponents assert that, when integrated into a broader behavioural intervention, it can serve as a trauma-informed response, providing safety and predictability [34,36]. This potentially highlights a role for post-incident review following such coercive practice exposures.

The remaining profiles describe scenarios where coercive practice is used in a goal-directed way to achieve compliance with medication or medical treatment (e.g., taking a blood sample). ‘Threat-compliance coercion’ involved implicit or explicit pressure from staff, which, while less invasive compared to an injection under physical restraint, likely involved the experience of threat and a loss of autonomy for the patient. This aligns with Hempeler et al.’s [37] model of informal coercion which highlights how the perceived likelihood of formal coercive measures (e.g., forced injection) influences patient behaviour. Corresponding with patient reports of experiences of post-incident reviews [25] this may suggest the delivery approach of intervention is a key consideration.

‘Passive refusal’ and ‘resistive refusal’, share a common function; administration of treatment medication by injection (e.g., anti-psychotic depot). These profiles are delineated however, by inclusion of additional coercive practices, e.g., rapid tranquillisation and seclusion present in the latter. This may reflect staff perceptions of anticipated risk of resistance or violence. While interpretations based on race/ ethnicity are limited due to missing data, consistent with wider literature, higher-restriction profiles (2–4) contained greater proportions of Black and racially minoritised patients. These findings reinforce concerns about the disproportionate impact of coercive practice on these groups.

### Post-incident reviews

The prevalence of post-incident review in this dataset (84%) aligns with the 93% rate reported by Asikainen et al. [19] but contrasts with qualitative studies suggesting post-incident reviews are not consistently implemented [32]. This variability in implementation rates may arise from inconsistency in post-incident review definitions, workload pressures, reporting burden, and concerns about accountability when reviews do not occur [15,20,33].

Profiles involving more invasive types of coercive practice (i.e., classes 2–4: threat-compliance coercion, passive refusal and resistive refusal) were associated with a lower occurrence of post-incident review. This may reflect more acute patient presentations which can increase the need for more intensive intervention and make post-incident review less feasible. This pattern could also signal a breakdown in trust between patients and staff. In this context coercive practice to enforce compliance becomes more probable further eroding trust and limiting opportunities for dialogue, such as a post-incident review [10,38]. Staff discomfort or ambivalence following highly restrictive interventions may also reduce the likelihood of initiating post-incident support [39]. In such situations, practices that promote dialogue and mutual understanding could be highly beneficial.

The lower likelihood of post-incident review in profiles that also contain higher proportions of Black and racially minoritised patients may reflect broader racial inequities in mental healthcare, e.g., greater likelihood of crisis-driven or aversive admission pathways [40]. The drivers for these disparities are complex with individual, systemic, and structural factors interacting to create and sustain racial inequities [41,42]. Among racially minoritised groups, fear, mistrust, and prior oppression often hinder help-seeking and access to treatment, resulting in more acute presentation and necessitating more service intervention [42,43]. These practices, serve to reinforce and further propagate negative perceptions of mental healthcare making post-incident reviews less tenable. Trauma-informed and anti-racism approaches, such as the patient carer race equality framework (PCREF), [44] could offer potential avenues to improve patient experiences and address these disparities. Advanced choice documents provide one example of how this approach could work in practice [45].

### Staff reported reasons for post-incident review non-occurrence

Coercive practice exposure profiles were significantly associated with staff-reported reasons for post-incident review non-occurrence. ‘Patient refusal to take part...’ was more likely in more invasive profiles (i.e., classes 2–4) potentially suggesting trust and staff-patient relationships as a key modulating factor in post-coercive practice support engagement. This may also indicate that post-incident reviews are not always the appropriate form of support or are being offered at the wrong time.

The higher likelihood of ‘other’ as the reported reasons for review non-occurrence in more invasive profiles highlights limitations in the available data regarding the underlying reasons for review omission. Furthermore, the higher likelihood of the reason being ‘not reported’ in more invasive profiles, reflects limitations in documentation of omissions. ‘Clinical decision to delay’ was not associated with coercive practice profiles suggesting a further gap in understanding staff decision-making processes around offering post-incident reviews. These could be useful targets of future research to inform intervention development and implementation.

### Limitations

The dataset was drawn from a single NHS mental health service provider which might limit generalisability of the findings and reflect local practices rather than national trends. The cross-sectional design and limited three-year study period, which coincided with the COVID-19 pandemic, may have introduced bias and further limited generalisability. Additionally, several terms used in the dataset to describe post-incident review practices are not explicitly defined in the providers policy, which may have led to inconsistencies and variation in reporting practices. Data quality, particularly the proportion of unreported patient ethnicity data, constrained interpretation. This is a critical limitation given the known associations between race/ethnicity and coercive practice. The unavailability of other covariates in the dataset, such as diagnosis and symptom severity, also constrained interpretation. Although evidence of associations of such factors and risk of exposure to coercive practice is not consistent across the literature, [46] it is possible the inclusion of further covariates may have refined interpretation.

It is also unclear whether staff record all types of coercive practice across the timeline of the incident or only the most restrictive interventions. If reporting focuses primarily on the most restrictive practices, earlier use of less invasive or restrictive practices may be underrepresented in the data. Furthermore, the missing data necessitated the exclusion of some data in the second stage of the latent class analysis (adding covariates), potentially introducing bias. The procedure for selecting the latent class analysis model and interpreting and labelling classes is also open to subjectivity and bias [30]. Class fallacy is a risk with all latent class analysis which could lead to misinterpretation of coercive practice experiences which then extend through the analyses. In the present study, efforts were made to mitigate this risk by validating model selection and class labelling with the LEAP and wider research team. A further qualitative investigation to triangulate the data and the findings would be beneficial.

### Implications

Viewing post-incident reviews as the sole support intervention after coercive practice is too narrow. Findings suggest relying on post-incident reviews may be insufficient for addressing the full breadth of patient support needs and highlight potential value in broadening the scope of post-coercive-practice support, tailoring the response to the type of coercive practice exposure.

The findings also highlight the influence of contextual and demographic factors on the delivery of post-incident reviews. This suggests clinicians should be cognisant of the context in which proceduralised interventions such as post-incident reviews are delivered. Addressing contextual factors, such as relational repair, may support the acceptability and effectiveness of proceduralised interventions. For patient groups with documented mistrust of mental health services, such as those from Black and racially minoritised backgrounds, [47] this will require racially and culturally informed practice. This may contribute to addressing the disparities identified in this study.

Trauma informed principles (e.g., empathy, recognition of the role of trauma in shaping mental health outcomes) [48] may offer a useful framework for strengthening post-coercive practice support. Integrating structured post-coercive practice support into existing coercive practices procedures may help operationalise this framework. Strengthening lived experience and community involvement in governance, as proposed by the anti-racism, Patient Carer Race Equity Framework, [44] could also be an impactful policy and implementation framework.

Further research should explore effective post-coercive practice support from the perspectives of those with lived experience, including Black and racially minoritised groups. There is also a need to understand the impact of omitted or declined support. Further studies should conceptualise coercion as an intersectional experience generated from the cumulative effects of coercive practices in specific contexts rather than focusing on discrete interventions, e.g., seclusion.

## Conclusion

This study provides novel insights into use of post-incident reviews following coercive practice in mental health inpatient settings. Findings indicate variation in the occurrence of post-incident reviews across coercive practice exposure profiles with reviews less likely to occur following exposure to more invasive coercive practices. Profiles associated with lower occurrence of post-incident reviews also contained a higher proportion of patients from Black and racially minoritised groups. While ethnicity was not independently associated with review occurrence, this observation highlights patterns that warrant further investigation. Broadening the scope of post-coercive practice support provision and considering how individual and contextual factors influence support could enhance patient experience and reduce inequities. Future research should further explore lived experience perspectives on post-coercive practice support, including those of groups more often represented in coercive practice exposure profiles with lower occurrence of post-incident reviews (i.e., Black and racially minoritised groups), to inform policy and practice improvements.

## Supporting information

S1 Text Table A. GRIPP-2 short form for reporting public and patient involvement. Table B. Exposures: Coercive practice categories and types. Table C: Model fit criteria. Table D. Diagnostic criteria. Table E. Gender, ethnicity and age composition of each latent class.
(DOCX)
