# Supplementary material for: More coercion, less support: A latent class analysis of post-incident reviews for mental health inpatients exposed to coercive practice
Source: PLOS Ment Health. 2026 Apr 15;3(4):e0000511. doi: 10.1371/journal.pmen.0000511 (PMC13082610; doi:10.1371/journal.pmen.0000511)
Supplement: S1 Text — Table A. GRIPP-2 short form for reporting public and patient involvement. Table B. Exposures: Coercive practice categories and types. Table C: Model fit criteria. Table D. Diagnostic criteria. Table E. Gender, ethnicity and age composition of each latent class. (DOCX) [file pmen.0000511.s001.docx]

**S1 Text**

**Table A**. GRIPP-2 short form for reporting public and patient involvement^1^

| **Section** | **Overview** |
| --- | --- |
| Aim | To ensure interpretation of latent class modelling was grounded in lived experience perspectives. |
| Method | A Lived Experience Advisory Panel (LEAP) was established to support the study. Membership comprised patients and informal (unpaid) carers (i.e. relatives) with direct or indirect experience of coercive practice in inpatient mental healthcare. Members were remunerated for their contributions. |
| Outcome | Consultations contributed to the interpretation, selection of the latent class analysis model, and naming of the classes in the final model. |
| Discussion | Consultations and engagements with the LEAP were integral to the selection and qualitative interpretation and naming of the latent class analysis model. |
| Reflection/Critical Perspective | Achieving meaningful engagement with a complex statistical output was challenging and required consideration for how the information was presented and the amount of time needed for the discussion. |

1. Staniszewska S, Brett J, Simera I, Seers K, Mockford C, Goodlad S, et al. GRIPP2 reporting checklists: tools to improve reporting of patient and public involvement in research. BMJ. 2017:j3453.

| Table B: Exposures: Coercive practice categories and types. | |
| --- | --- |
| Category | Type |
| Physical restraint | Standing |
|  | Restricted escort |
|  | Kneeling |
|  | Seated |
|  | Supine |
|  | Prone |
|  | Side |
|  | SafetyPod |
|  | Standing |
| Enforced treatment | Prescribed medication - mental illness |
|  | Prescribed medication- physical illness |
|  | Medical intervention |
| Chemical restraint | Oral |
|  | Rapid tranquilisation |
|  | Injection not rapid tranquillisation |
|  | Other |
| Not applicable | Mechanical restraint |
| Not applicable | Seclusion |
| Not applicable | Segregation |
| Not applicable | Tear-proof clothing |

| **Table C:** Model fit criteria | | | | | | | |
| --- | --- | --- | --- | --- | --- | --- | --- |
| **nclass** | **nsample** | **Observed cases** | **Npara-meters** | **LL** | **AIC** | **BIC** | **SABIC** |
| 1 | 8263 | 8170 | 19 | -48873.90 | 97785.81 | 97919.18 | 97890.62 |
| 2 | 8263 | 8170 | 39 | -46466.24 | 93010.49 | 93284.25 | 93518.78 |
| 3 | 8263 | 8170 | 59 | -45710.07 | 91538.14 | 91952.30 | **92750.56** |
| 4 | 8263 | 8170 | 79 | -45304.91 | 90767.82 | 91322.36 | 92985.03 |
| 5 | 8263 | 8170 | 99 | -45010.02 | 90218.04 | 90912.97 | 93740.70 |
| 6 | 8263 | 8170 | 119 | -44789.03 | 89816.06 | 90651.38 | 94944.83 |
| 7 | 8263 | 8170 | 139 | -44519.63 | **89317.26** | **90292.97** | 96352.80 |

| **Table D.** Diagnostic criteria | | | | | |
| --- | --- | --- | --- | --- | --- |
| **nclass** | **Smallest class count (n)** | **Smallest class size (%)** | **LMR LR** | **BLRT** | **Entropy** |
| **1** | 8263 | 100 | - | - | - |
| **2** | 3751 | 45.40 | 0.000 | 0.000 | 0.746 |
| **3** | 1237 | 14.97 | 0.000 | 0.000 | **0.929** |
| **4** | 685 | 8.29 | 0.000 | 0.000 | **0.822** |
| **5** | 644 | 7.79 | 0.000 | 0.000 | 0.758 |
| **6** | 586 | 7.09 | 0.000 | 0.000 | 0.656 |
| **7** | 603 | 7.30 | 0.000 | 0.000 | 0.733 |

| **Table E.** Gender, ethnicity and age composition of each latent class | | | | | | | | | | | | |
| --- | --- | --- | --- | --- | --- | --- | --- | --- | --- | --- | --- | --- |
|  | **Class 1** | | | **Class 2** | | | **Class 3** | | | **Class 4** | | |
| **Characteristic** | **n** | **%** | **95% CI** | **n** | **%** | **95% CI** | **n** | **%** | **95% CI** | **n** | **%** | **95% CI** |
| **Gender** |  |  |  |  |  |  |  |  |  |  |  |  |
| Female | 2031 | 81.763 | [80.084 - 83.443] | 676 | 31.095 | [27.605 - 34.584] | 508 | 46.907 | [42.567 - 51.246] | 1212 | 57.035 | [54.248 - 59.822] |
| Male | 453 | 18.237 | [14.681 - 21.793] | 1287 | 59.200 | [56.515 - 61.885] | 575 | 53.093 | [49.014 - 57.172] | 913 | 42.965 | [39.754 - 46.176] |
| Not specified | 0 | 0.000 | - | 211 | 9.706 | [5.711 - 13.700] | 0 | 0.000 | - | 0 | 0.000 | - |
| **Ethnicity** |  |  |  |  |  |  |  |  |  |  |  |  |
| Not recorded | 631 | 25.403 | 22.006 - 28.799 | 322 | 14.811 | [10.932 - 18.691] | 206 | 19.021 | [13.662 - 24.381] | 411 | 19.341 | [15.523 - 23.160] |
| Asian | 7 | 0.282 | [-3.645 - 4.209] | 51 | 2.346 | [-1.808 - 6.500] | 26 | 2.401 | [-3.483 - 8.285] | 57 | 2.682 | [-1.512 - 6.877] |
| Black | 174 | 7.005 | [3.212 - 10.797] | 443 | 20.377 | [16.626 - 24.128] | 250 | 23.084 | [17.861 - 28.307] | 317 | 14.918 | [10.996 - 18.840] |
| Mixed | 158 | 6.361 | [2.555 - 10.166] | 75 | 3.450 | [-0.681 - 7.580] | 19 | 1.754 | [-4.149 - 7.658] | 97 | 4.565 | [0.411 - 8.718] |
| Not stated | 664 | 26.731 | [23.365 - 30.097] | 521 | 23.965 | [20.300 - 27.631] | 163 | 15.051 | [9.561 - 20.540] | 387 | 18.212 | [14.367 - 22.057] |
| Other | 69 | 2.778 | [-1.100 - 6.655] | 493 | 22.677 | [18.981 - 26.374] | 294 | 27.147 | [22.063 - 32.230] | 700 | 32.941 | [29.459 - 36.423] |
| White | 781 | 31.441 | [28.185 - 34.697] | 269 | 12.374 | [8.439 - 16.308] | 125 | 11.542 | [5.940 - 17.144] | 156 | 7.341 | [3.248 - 11.434] |
| **Age** |  |  |  |  |  |  |  |  |  |  |  |  |
| Mean (SD) |  | 15.728 (1.379) | |  | 33.464 (15.499) | |  | 41.877 (16.071) | |  | 36.943 (13.906) | |
| Median |  |  | 16 |  |  | 30 |  |  | 39 |  |  | 35 |
| Minimum |  |  | 4 |  |  | 13 |  |  | 15 |  |  | 15 |
| Maximum |  |  | 18 |  |  | 90 |  |  | 89 |  |  | 87 |
| Lower Quartile |  |  | 15 |  |  | 22 |  |  | 29 |  |  | 26 |
| Upper Quartile |  |  | 17 |  |  | 41 |  |  | 54 |  |  | 46 |
| IQR |  |  | 2 |  |  | 19 |  |  | 25 |  |  | 20 |
